# An Assessment of Daily Energy Expenditure of Navy Ship Crews and Officers Serving in the Polish Maritime Border Guard as an Indicator of Work Severity and Nutritional Security

**DOI:** 10.3390/nu17060953

**Published:** 2025-03-09

**Authors:** Jerzy Bertrandt, Mieczysław Pawlisiak, Izabela Bolczyk, Tomasz Grudniewski, Roman Lakomy, Andrzej Tomczak, Karolina Bertrandt, Tomasz Lepionka, Dorota Brewinska, Justyna Bandura, Anna Anyzewska

**Affiliations:** 1 Faculty of Economic Sciences, John Paul II University in Biala Podlaska, Sidorska 95/97, 21-500 Biala Podlaska, Poland; 2 Faculty of Security, Logistics and Management, Military University of Technology, gen. Sylwestra Kaliskigo 2, 00-908 Warsaw, Poland; mieczyslaw.pawlisiak@wat.edu.pl; 3 Medical Department of the Technology and Supply Office of the Border Guard Headquarters, al. Niepodległości 100, 00-514 Warsaw, Poland; ibipolo@gmail.com; 4 7th Navy Hospital in Gdansk, ul. Polanki 117, 80-305 Gdańsk, Poland; tomek_doc@op.pl; 5 Independent Researcher, ul. Postepu 6B, 05-506 Lesznowola, Poland; roman.lakomy@interia.pl; 6 Institute of Human Sciences, WSB Merito University in Torun, ul. Mlodziezowa 31a, 87-100 Torun, Poland; 7 Military Institute of Aviation Medicine, Krasinskiego 54/56, 01-755 Warsaw, Poland; karolinabertrandt@gmail.com; 8 Laboratory of Next Generation Sequencing, Biological Threats Identification and Countermeasure Centre, Military Institute of Hygiene and Epidemiology, Lubelska 4, 24-100 Pulawy, Poland; tomasz.lepionka@wihe.pl; 9 Institute of Health Psychology of the Polish Psychological Society, Geslarska 3, 02-412 Warsaw, Poland; psychoterapiadb@wp.pl; 10 The 20th Military Spa and Rehabilitation Hospital, Swidzinskiego 4, 33-380 Krynica Zdroj, Poland; dietetyk.bandura@gmail.com; 11 School of Medical & Health Sciences, University of Economics and Human Sciences in Warsaw, Okopowa 59, 01-043 Warsaw, Poland; a.anyzewska@vizja.pl

**Keywords:** energy expenditure, obesity, navy, border guard, workload, nutrition security

## Abstract

Background: Research on energy expenditure related to life and professional activity allows us to determine a person’s energy. This study determined the value of daily energy expenditure related to the implementation of service and training tasks of crews of ships in the Polish Navy and officers of the Maritime Department of the Border Guard. Materials and Methods: This study included crews of two selected ships of the Polish Navy and officers of the Maritime Branch of the Border Guard. The method of analyzing heart rate changes was used to measure energy expenditure. Results: The value of daily energy expenditure related to the implementation of tasks during a cruise amounted to 3874 kcal/d for the crew of a missile frigate, and it was higher at 4031 kcal/d for the crew of a training sailing ship. Energy expenditure related to the training of ship crews staying in a port was lower, amounting to 3648 kcal/d and 3380 kcal/d, respectively. The energy expenditure of the Maritime Border Guard officers during a 12 h shift ranged from 1830 kcal/12 h to 2762 kcal/12 h. Current nutritional standards for sailors of the Polish Navy and the Border Guard officers provide an excessively high energy intake in daily food rations, which may cause overweight and obesity.

## 1. Introduction

The state of human health depends on many factors that influence a body throughout life, shaping its optimal physical and mental well-being. The term “food safety” refers directly to food, while nutrition security relates to access to essential nutrients, not food in general. Nutrition security means ensuring that available food enables the achievement of nutritional goals. Such goals nowadays include not only providing energy and nutrients to meet the body’s physiological needs and physical activity levels but also supporting a health-promoting nutrition model by ensuring the availability of products that are more beneficial to health. In other words, nutrition security is not only about meeting the nutritional needs of humans resulting from their physiological needs and physical activity but also about an appropriate, health-promoting nutrition model that prevents nutritional disorders, especially those related to overfeeding [1,2,3].

This study aims to assess the value of daily energy expenditure related to the service and training tasks of crews from two selected Polish Navy ships, as well as officers of the Maritime Border Guard Unit, as a factor influencing nutrition security and work safety.

Research regarding the personnel of these two formations is justified due to the significant similarity of the tasks they perform, which include, among others, the protection of inviolability of the maritime border and the detection of symptoms of threats to state security from the sea, the protection of the Polish economic zone, the protection of important communication routes, participation in search and rescue operations at sea (SAR), and participation in the ecological protection of Polish maritime areas, as well as the detection of pollution of the marine environment and detecting perpetrators [4,5].

This study was carried out to develop nutrition standards and dietary recommendations for the Polish Navy ship crews and officers of the Border Guard Maritime Department. Currently, ship crews are fed based on food standards, which define the energy and nutritional value of food rations based on the values of individual products used in the planning and implementation of nutrition and not on the values of actual energy expenditure related to the nature and specificity of the service. For the planning and implementation of nutrition of the Border Guard officers, there are no nutritional norms. Therefore, it was purposeful to determine the real energy expenditure of the Navy ship crews and officers serving in the Border Guard Maritime Department as a basis for the development of nutrition norms or the verification of used food standards. Taking into account the occurrence of overweight and obesity among Polish Navy ship crews found in the previous long-term research, it should be assumed that the energy value of the food standard used in planning and implementation is too high and has caused a positive energy balance [6,7]. The second aim was to determine a workload of crews serving on selected Polish Navy ships and officers of the Border Guard Maritime Unit, which will allow for a rational dosage of workload in the training process of sailors and officers according to their physical capabilities.

The research was conducted by the interdisciplinary team of employees from various scientific institutions, including doctors, nutritionists, dieticians, sports physiologists, and psychologists.

## 2. Materials and Methods

### 2.1. Participants

The energy expenditure examination comprised 104 males from the crews of two Polish Navy ships: the missile frigate, which has 74 people, and the Polish Naval Academy’s training sailing ship, which has 30 people. Additionally, 110 officers of the Border Guard Maritime Branch took part in this study, including 21 from the Kashubian Maritime Border Guard Division. It should be mentioned that officers from the Kashubian Maritime Border Guard Division comprised a permanent crew of the Border Guard ship, while others were on the ship temporarily, specifically only during the fulfillment of their duties. Due to the small number of women serving on Navy ships and in the Border Guard Maritime Unit, the research included only male participants.

The research was conducted as part of typical training and military service planning activities. For Navy ship crews, the study included training at sea during sea voyages and in a port. For Border Guard officers, the study assessed energy expenditure during the implementation of tasks in a typical day of work and service.

The research was conducted in accordance with the Helsinki Declaration of the World Medical Association. It was approved by the Ethics Committee of the Military Institute of Hygiene and Epidemiology (no. 1/XXI 95/2016). Participants received an information sheet about the study details, purpose, procedures, and potential risks and benefits of their participation. All participants provided informed consent.

### 2.2. Measurement of Height and Weight

Body height (without shoes) was measured using a portable stadiometer (TANITA HR-001, Tanita Corporation, Tokyo, Japan). Body weight was measured using bioelectrical impedance analysis (BIA) with the TANITA MC-780 103 device (Tanita Corporation, Tokyo, Japan), with an accuracy of 0.1 kg, according to the procedure specified in the instruction manual (lightly dressed and without shoes). All measurements were performed according to the procedure specified in the instruction manual and without any metal objects.

### 2.3. Measurement of Energy Expenditure

This study used the method of analyzing heart rate changes to measure energy expenditure. This is the only possible method to measure energy expenditure during the performance of service and combat tasks on board, as well as during the performance of duties by the Border Guard officers. The method is based on a linear relationship between heart rate (HR) and oxygen uptake (VO_2_) during exercise. The energy expenditure examination included heart rate measurements with a Polar RC3 GPS heart rate monitor (Polar Electro Oy, Kempele, Finland) [8]. Polar watches are acceptable for estimating daily energy expenditure during activities performed across various intensities and are practical for use in the field [9]. This watch calculates the energy expenditure value from the relationship between heart rate and the energy cost of the work, in accordance with the ISO 8996:1990E [10], and the result is an average of individual measurements. Previous research has shown that these devices have a relatively high accuracy, and measurements taken in healthy subjects are like those taken in parallel with electrocardiograms (correlation coefficient r = 0.99) [11]. In addition, a validation of devices was carried out using the MWE-1 energy expenditure meter by CB electronics, in which the principle of the energy expenditure assessment is based on the measurement of the forced expiratory airflow of an examined person, as well as the correlation found between the ventilation volume and oxygen uptake, thus providing the energy expenditure [12]. Before starting the study, heart rate and VO_2_ calibration curves were determined for each subject. The values of energy expenditure associated with the typical activities of daily life on a ship and in the Border Guard Maritime Unit, such as sleeping, washing oneself, cleaning, physical exercise, eating, watching television, and others, were taken from the tables of energy expenditure of Polish soldiers serving in different types of troops and services [13]. The research was carried out for over 6 months, both during the training of ship crews at sea and during service activities in a port. Similarly, as on Navy ships, the examination of Border Guard Maritime Unit officers was conducted for 6 months and included activities at sea as well as in a port. The obtained results were used to determine the energy demand in relation to the specificity and nature of the service and to assess the intensity of the work in accordance with the classification given by Lehman or Christensen (Table 1 and Table 2) [14,15].

### 2.4. Statistical Analyses

All statistical analyses were performed using the R program (the R Foundation for Statistical Computing v2.0–1. https://cran.r-project.org (accessed on 20 September 2021)). Anthropometric data are shown as mean values ± standard deviation, and differences between experimental groups were analyzed with the Anova and Tukey tests, which were statistically significant when *p* < 0.05. To check the compliance of variables with a normal distribution, the Shapiro–Wilk test was used, and Levene’s test was used to verify the homogeneity of variance. Differences in energy expenditure values between Navy ship crews and Border Guard officers were calculated using the chi-square test.

## 3. Results

### 3.1. Characteristics of the Study Groups

Before starting the study, anthropometric measurements were taken of all the people participating in the study. The characteristics of the study participants are presented in Table 3.

### 3.2. Daily Energy Expenditure of Selected Navy Ships Crews During Training at Sea

The examination of the energy expenditure of ship crews at sea was carried out during training cruises lasting several weeks. The research included 74 sailors of a missile frigate and 30 students of the Naval Academy who were a crew of a training sailing ship. The participants performed typical everyday activities related to serving on a ship in various positions. The values of energy expenditure while performing activities on board and daily energy expenditure are summarized in Table 4 and Table 5.

According to Christensen’s classification of work severity, the value of energy expenditure of individual activities performed by a ship’s crew during a cruise should be classified into categories ranging from light to hard work. The average daily energy expenditure of the missile frigate’s crew, resulting from the implementation of training tasks and operating a ship at sea, amounted to 3874 kcal, which allows the work performed by these sailors to be classified as hard work. On the training sailing ship, average energy expenditure values characterizing typical activities performed during a training cruise ranged from 2.17 to 5.61 kcal/min. Based on Christensen’s classification of work severity, these activities can be classified as light to moderate work. However, the average daily energy expenditure during training activities at sea for the sailing ship crew was approximately 4032 kcal, which also falls into the category of hard work. The average values of energy expenditure characterizing typical activities performed during a training cruise on the sailing ship ranged from 2.17 to 5.61 kcal/min, which, according to Christensen’s classification of work severity, can be classified as light to moderate work, while the average daily energy expenditure value during training activities at sea was about 4032 kcal, which allows the work performed to be classified as hard work.

### 3.3. Daily Energy Expenditure of Selected Navy Ship Crews During Stay in a Port

When a ship is docked in a port, a crew performs maintenance and training tasks according to the port’s training plan.

The average daily energy expenditure values for a missile frigate and a training sailing ship crews during their stay in a port are summarized in Table 6 and Table 7.

The value of the daily energy expenditure of the missile frigate’s crew during the dock was 3648 kcal/day, which qualifies the work performed as moderate hard work. Although the daily energy expenditure of the cadets was lower (3380 kcal) on the days of their stay in a port, the work performed on those days could also be classified as moderate hard work.

### 3.4. Energy Expenditure of Border Guard Officers Related to the Service and Implementation of Training Tasks

The Border Guard Unit supervises sea border crossings, while the Kashubian Border Guard Division supervises the exploitation of Polish maritime areas and the compliance of ships with the regulations enforced in these areas. The energy expenditure of Border Guard officers was measured during a 12 h work shift. The energy expenditure examination included 89 officers of the Maritime Border Guard Unit and 21 members of the crew of the Kashubian Border Guard Division’s ship. Energy expenditure values per work shift are summarized in Table 8 and Table 9.

An analysis of the results showed that the average energy load of Border Guard officers performing official tasks in the Border Guard Unit and Kashubian Border Guard Division amounted to 1703 ± 599/12 h and 1178 ± 462/12 h, respectively. The average energy expenditure of officers per 8 h, i.e., per work shift, amounted to 1469 kcal, which allows it to be classified as medium work according to the Lehman classification of work severity [14].

### 3.5. Energy Value and Content of Protein, Fats, and Carbohydrates in Rations Planned and Given for Consumption to Sailors of Selected Polish Navy Ships

The energy value and the protein, fat, and carbohydrate content of planned and given for consumption food rations in relation to the food standard are summarized in Table 10.

The analysis of obtained results allowed us to conclude that the average energy values of the food rations both planned and given for consumption to the crew of a rocket frigate were lower by more than 101 and 412 kcal, respectively, than the energy value of food rations provided in the food standard. The protein content of the planned food ration was also lower, which was exacerbated in the ration given for consumption and was significantly lower than the food standard by 15.3 g, representing 9.3% of the total protein supply. A fundamental error in menu planning was the excessively high fat content of 201 g, which exceeded the food standards by more than 56 g. The fat content of the food ration given for consumption to the sailors was 147.4 g, which was only 2.1 g higher than the standard. A large difference found in the fat content between planned rations and the ones given for consumption indicates the inadequate implementation of menus planned by the food service staff. Also, the carbohydrate content of both planned and given for consumption food rations was significantly lower than the military food standard [16].

The evaluation of the school sailing ship crew’s nutrition revealed that the energy value of planned food rations was too low in relation to the food standard, possibly due to insufficient carbohydrates content. While the protein content was within the limits of the food standard, planned fat supply was too high and exceeded the standard by more than 22 g. Irregularities in food planning are reflected in the energy and nutritional value of food rations given for consumption. Their analytically determined energy value was more than 1000 kcal lower than the applicable food standard, which accounted for 22.3% of the total energy value. This was due to insufficient content of all macronutrients. Profound deficiencies shown in the energy value and in the protein, fat, and carbohydrate content of the rations given for consumption were due to poor nutritional planning, but mainly due to the inadequate implementation of nutrition on board by food service personnel.

### 3.6. Percentage of Energy from Protein, Fats, and Carbohydrates in Food Rations Planned and Given for Consumption to the Sailors of Selected Polish Navy Ships

**Figure 1:**
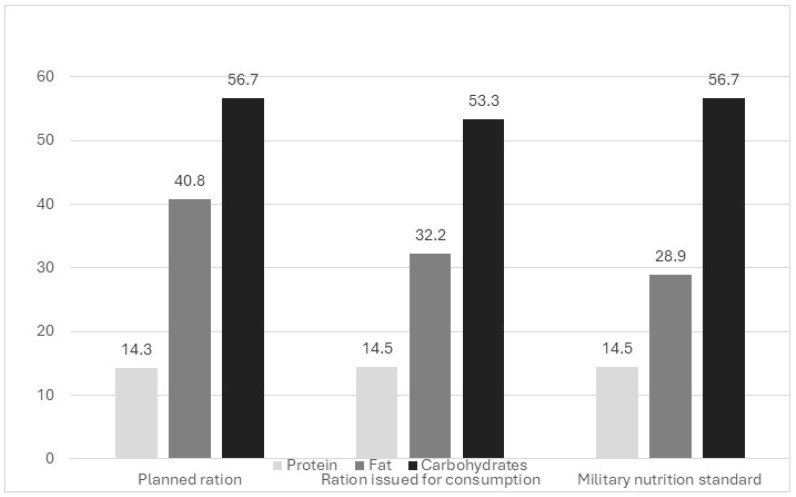
Percentage of energy from proteins, fats, and carbohydrates in an average food ration planned and given for consumption to the rocket frigate’s crew.

**Figure 2:**
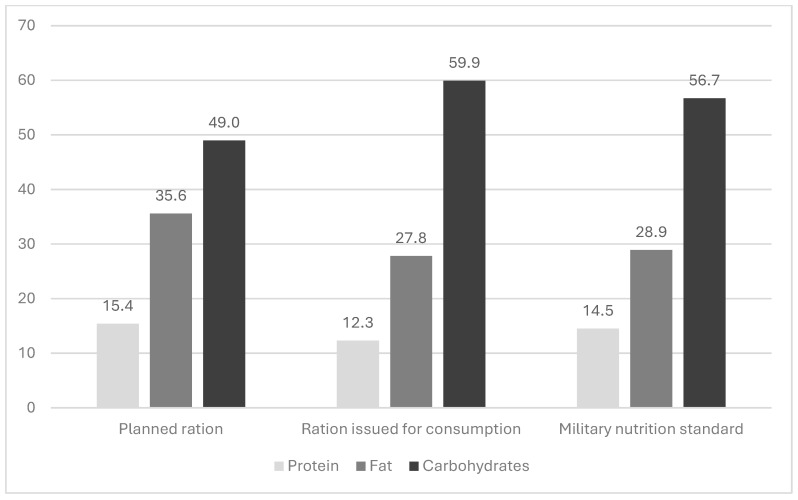
Percentage of energy from proteins, fats, and carbohydrates in an average ration planned and given for consumption to the school sailing ship’s crew.

The percentage of energy derived from proteins, fats, and carbohydrates in food rations planned and given for feeding ship crews is shown in Figure 1 and Figure 2.

Data presented in Figure 1 show that too high a percentage of energy came from fats and too low a percentage from carbohydrates in both planned and consumed food rations. It should be noted that in the Polish Army, there are standards of nutrition depending on the types of troops; the standards are adapted to the type of service and are associated with its energy burden. In the analyzed food rations, inconsistencies, in comparison with the obligatory norms, occurred already at the planning stage. Planned food rations contained too much fat and too little carbohydrates, which is a direct cause of incorrectness shown in rations given for consumption.

The analysis of the energy value of individual macronutrients in the ration planned for feeding the school sailing ship’s crew showed an excessive supply of proteins and fats, with an inadequate supply of carbohydrates in relation to the food standard. Apart from the fact that the energy value of food rations given for consumption was significantly lower and did not cover the values provided for in the food standard, there was a shortage of energy from proteins and fats, with a slight oversupply of energy from carbohydrates, which significantly disturbed the correct proportions of energy from individual macronutrients.

Considering the obtained results, it must be concluded that both the planning and implementation of nutrition to school sailing ship crews were incorrect. These irregularities resulted in a low energy value of daily food rations and too low contents of proteins, fats, and carbohydrates. This feeding pattern did not cover the daily energy expenditure and did not provide adequate amounts of macronutrients, which may be a cause of a negative daily energy balance. This may result in weight loss and a decrease in the physical performance of the sailors.

Because there are no nutrition standards for Polish Border Guard officers and nutrition is provided only based on a defined financial limit, no research was conducted on the energy and nutritional value of food rations. Research on the energy expenditure of officers of the Border Guard Maritime Unit related to the service and task performance was undertaken for the first time for this formation, and results will constitute the basis for the development of a nutrition norm by considering the specificity and nature of the work performed.

## 4. Discussion

Being overweight or obese, which is a result of a positive energy balance resulting from a faulty, energy-rich diet and low physical activity, not only worsens one’s well-being but also constitutes health and social problems associated with limiting the possibility of a soldier’s profession and/or Border Guard officer [17,18].

The available literature indicates that overweight and obesity are significant health problems in many armies worldwide [19,20,21,22]. Obesity is also a problem in the Polish Army. It has been shown that in a group of 355 soldiers in one Polish Army Unit, a percentage of fatness of >20% was found among 46% of the examined subjects [23]. This is probably related to the high stress levels and harmful environmental factors of military service, especially during exercises, military missions, or during deployment and relocation [24,25,26]. Factors such as the type of armed forces, BMI, the place of residence, and the level of education also play an important role [27]. In addition, there may be restrictions on the choice or availability of food, particularly for certain services such as the navy [28].

The prevalence of body mass disorders among navy crews found in the present study confirms the results of earlier studies, which showed that the number of overweight sailors increased with the age of the subjects. Overweight was indicated among 49% of ship crews aged up to 30 and 54.3% of sailors aged up to 40 [6]. The results of the assessment of energy expenditure obtained in this study are a confirmation of previous research, in which it was shown that the value of the energy expenditure of activities performed by crews of various Polish Navy ships, both during exercises at sea and during the ship’s stay in a port, varies greatly and can be classified in categories ranging from light to extremely heavy work [29].

The results of the examinations of energy expenditure of Polish Navy ship crews are similar to the results of previous research conducted in other armies.

The analysis of the results of many studies conducted in the armies of different countries has led to the conclusion that the energy expenditure of military personnel, soldiers serving in land forces, air forces, the navy, and special forces, as measured in a garrison and during field training under different climatic conditions and in different activities, ranges from 13.0 to 29.8 MJ (3109–7131 kcal)/day [30].

Service on navy vessels is highly specific, not only due to the nature of the performed training tasks but also due to the restricted environment of a ship and the influence of adverse external conditions resulting from being at sea. Previous examinations of energy expenditure of crews of various types of Polish Navy vessels showed that the average value of daily energy expenditure during training in a port was 4000 kcal, while at sea, it ranged from 4200 to 4700 kcal, depending on the type of a ship and the nature of the performed training tasks [31]. In comparison, the results of the energy burden of Australian sailors, published in 1990, showed that they were burdened with an energy expenditure of 13,850 ± 2510 kJ (3305 ± 599 kcal) over seven days of training [32]. Energy expenditure studies conducted in the Indian Navy have shown that service on ships generates a daily energy expenditure of 3313 ± 578 kcal/day for a ship’s crew, while for a submarine’s crew, it is 3168 ± 282 kcal/day [33].

Another research showed that during deployment, submarine crews’ total daily energy expenditure amounted to 3315 ± 560 kcal/d and 3078 ± 413 kcal [34,35].

A high daily energy expenditure of 19.7 MJ (4725 kcal) was also indicated among women beginning service and training in the US Navy during a 54 h basic training session. Their total energy expenditure was lower than that of men, which amounted to 25.5 MJ (6085 kcal) [36]. Daily energy expenditure was also assessed during two weeks of conventional basic training for sailors. This was significantly lower and amounted to 9.9 MJ (2663 kcal) for women and 16.9 MJ (4033 kcal) for men [37].

Results of the study by Tharion et al. on the energy burden of US Navy sailors trained at sea showed that the daily energy expenditure for women was 11.6 ± 1.8 MJ (2766 kcal) and for men amounted to 14.4 ± 3.6 MJ (3446 kcal) [38]. Research carried out in the Australian Navy showed that serving and schooling male sailors were burdened with an energy expenditure of 3310 kcal/day [39]. An assessment of daily energy expenditure of sailors serving in a naval base showed that their daily energy expenditure averaged 3305 ± 599 kcal, while for the physically active group, it was 3830 ± 286, and for the inactive group, it was 2823 ± 387 [39].

In the Royal Navy, the average daily energy expenditure of sailors undertaking general ship-board duties at sea is estimated to be 3389 ± 731 kcal and 2356 ± 440 kcal in men and women, respectively [40]. These values were consistent with previous estimates that amounted to 3391 ± 635 kcal and 2393 ± 700 kcal in male and female sailors, respectively, based on measurements taken during basic operational training at sea [41].

The nutritional requirements of navy sailors differ from those of ground forces because they work in a closed environment and because of the logistical constraints associated with cooking and storing food. Therefore, it is very important to ensure the supply of appropriate energy through food rations to balance the energy expenditure of navy ship crews. On the other hand, an adequate supply of macronutrients, i.e., proteins, fats, and carbohydrates, in terms of both quantity and appropriate proportions plays an important role. The present study showed a disproportion between the supply of proteins, fats, and carbohydrates in both planned and given for consumption rations, which disturbed the correct proportions between them. Previous examinations of rations used in the nutrition of Polish soldiers indicated this problem [42]. These disproportions are due to Polish dietary habits, which favor the use of large amounts of fats in meal preparation technology, as well as increasing the energy value of food rations by adding fats. For comparison, the energy value of rations used in the nutrition of Indian Navy ship crews amounted to 3518 ± 286 kcal/day, with the proportion of energy coming from carbohydrates, fats, and proteins being 59.9%, 27.8%, and 12.3%, respectively [33].

The available literature did not find any studies on the energy burden of Border Guard officers. Officers of the Polish Border Guard are fed individually and not always rationally, which may not meet nutrition security criteria. In previous research, Anyzewska showed correlations between diet, physical activity, body mass index, fat mass index, visceral fat level, and bone mineral density in male Border Guard officers. Higher body mass and fat mass were correlated with poorer dietary habits, i.e., the low consumption of fruits, vegetables, dairy, nuts, grains, as well as with lower leisure-time physical activity and longer time spent sitting during the day [18].

In summary, the values of the daily energy expenditure of the Polish Navy ship crews obtained in the present study were similar to the results of previous studies conducted among crews of warships of other countries.

The excessive body mass shown in the research is a result of an unbalanced energy balance and results from too high a supply of energy from food in relation to the energy needs associated with schooling and service on ships, as well as the daily physical activity of sailors and Border Guard officers. The energy value of the food standard used in the planning and implementation of the nutrition of Polish Navy ship crews amounts to 4520 kcal, which exceeds the energy needs of sailors and contributes to the formation of overweight and obesity. For comparison, the standard for the energy value of food rations used in the nutrition of US Army soldiers is 3400 kcal/d for men and 2300 kcal/d for women, with a possibility of its increase to 3600 kcal/d under operational conditions [43].

Problems of being overweight and obesity occurrence are also observed among naval personnel in other armies in the world. The results of the examination of nutritional status of British sailors showed that 28% of Royal Navy (RN) personnel were exposed to increased risk of obesity development [44]. Results of other studies on the prevalence of obesity among Royal Navy staff showed that 23% of men and 37% of women, respectively, were at risk of obesity-related diseases [45]. This risk increases with age [46]. A study of a random sample of 1596 Royal Navy personnel showed that 13% were obese, and 42% were overweight [47].

A study to determine the frequency of obesity occurrence among Royal Navy personnel found that in a random sample of 1596 people, 13% were obese, and 42% were overweight [48]. Results of other examinations of 600 RN personnel aged 18–61 years serving in 15 land bases and on two ships showed that 65% of men and 44% of women were overweight or obese [48]. In the US Navy, a study of the nutritional status of 462 male submarine crewmen showed that the mean BMI and the body fat percentage of the sailors were 28.8 ± 4.1 and 28.9 ± 6.6%, respectively, and the percentage of body fat increased with age. The prevalence of obesity of varying degrees was demonstrated in 61% of the subjects [49]. Results of a cross-sectional examination of ship crews’ nutrition planning, implementation, and nutritional status involving 26,341 US Navy sailors showed the occurrence of obesity in 15.1% of personnel serving on aircraft carriers, in 16.9% of small submarine crews, and in 17.8% of large submarine sailors [50].

Results of the largest nutritional status assessment research, conducted in 2012 and covering a population of 313,513 US Navy sailors, showed the occurrence of obesity in 13.6% of those surveyed, with obese men accounting for 15.4% and women 4.6% [51].

Similarly, a study of the nutritional status of 16,365 US Navy personnel serving on missions in Iraq and Kuwait between 2005 and 2008 found that the body weight of 10,886 men was normal before the mission but increased significantly at the end of the mission.

This increase may have been due to a few factors, including free access to high-calorie foods, a lack of opportunity for exercise, or the stress/fear of loss of health and life that in-crease food intake [52].

Also, results of a nutritional status survey conducted in the Irish Navy showed that 48.6% of 820 examined sailors were overweight, and 16% were obese. It was also found that in the age group of 18–35, 58.4% of examined person were overweight or obese; among persons aged 36–50, 78% were overweight, and among persons aged 51–60, 95.6% were overweight or obese [53]. It was shown that the number of people who are overweight or obese increased with age.

The reasons for obesity among Navy personnel represent a kind of vicious circle. Fatigue resulting from physical strain associated with service can lead to lack of desire to exercise on board, which reduces the value of energy expended. In turn, a lack of exercise and the excessive energy value of food can lead to a positive daily energy balance, consequently resulting in obesity.

In conclusion, indicated nutritional disorders associated with excessive body weight of Polish Navy ship crews corroborate the results of studies conducted among personnel serving in the navy of other countries, which allows us to conclude that the obesity pandemic occurring worldwide also affects the military population, including naval personnel of many armies.

The results of the examination of the nutritional status of Polish Navy ship crews, as well as data concerning overweight and obesity occurrence among personnel serving in other armies, suggest the need to promote nutritional education, which will raise the awareness of rational, health-promoting nutrition to a higher level and, consequently, make correct nutritional choices.

However, this study has several limitations. The first limitation is the relatively small sample of respondents due to the full-time employment of ship crews and the small number of Border Guard officers carrying out control tasks at sea. Another limitation is the examination of only two selected Polish Navy ship crews and the specificity of the tasks they perform. It should be assumed that the results of research on the energy expenditure of crews of other types of ships will be significantly different; hence, the development of a unified standard of nutrition dedicated to Polish Navy ships crews requires further research. The research period is also a limitation. This study was conducted in the spring–summer period, while the working environment on a ship in the winter season may significantly affect the energy expenditure of crews and shape energy and nutritional needs. The lack of information about the energy and nutritional value of food eaten outside the ship is also a major limitation of nutritional status surveys. Demonstrated disturbances in the nutritional status of sailors and border guards are not only due to the used collective feeding model but also due to additional individual intake.

## 5. Conclusions

In conclusion, this study showed the following:

1. The energy load of Polish Navy ships crews and the Border Guard Maritime Unit officers related to the execution of official and training tasks varies and depends on the type of ship, the function performed on the ship, and the conditions for the performance of service and training tasks, and the severity of the work performed by an individual sailor is classified in categories ranging from light to very heavy;
2. The prevalence of overweight and obesity among ship crews and the Border Guard Maritime Unit officers requires the development and implementation of a dedicated dietary standard for these services, and it needs to be balanced in terms of energy and all nutrients;
3. There is a need to spread nutrition education among sailors, as well as personnel responsible for planning and implementing nutrition on ships, and among Border Guard officers in terms of the knowledge of a rational, health-promoting model of nutrition and, consequently, in making the right dietary choices;
4. Ensuring the nutritional safety of the Polish Navy crews and the Border Guard Maritime Unit officers requires the development and implementation of a nutrition model considering the nature and specificity of their service.

## Figures and Tables

**Table 1:** Classification of work severity based on energy expenditure values [14].

Degree of Severity of Work	Daily Energy Expenditure—24 h		Energy Expenditure Per Work Shift—8 h	
	Kcal	MJ	Kcal	MJ
Light	2300–2800	9.6–11.7	≤500	≤2.1
Moderate	2800–3300	11.7–13.8	500–1000	2.1–4.2
Moderate hard	3300–3800	13.8–15.9	1000–1500	4.2–6.3
Hard	3800–4300	15.9–18,0	1500–2000	6.3–8.4
Very hard	4300–4800	18.0–20.1	2000–2800	8.4–11.7
Extremely hard	≥4800	≥20.1	≥4800	≥11.7

**Table 2:** Classification of work severity based on energy expenditure in kcal/min [15].

Degree of Work Severity	The Amount of Energy Load
Light	>2.5 kcal/min
Moderate	>5.0 kcal/min
Hard	>7.5 kcal/min
Very hard	>10.0 kcal/min
Extremely hard	>12.5 kcal/min

**Table 3:** The characteristics of the study participants.

Ship	Misle Frigate No-74	Training Sailing Ship No-30	Maritime Border Guard Unit No-89	Kashubian Border Guard Division No-21	Average
Age [years]	29.8 ± 4.2	21.6 ± 2.8 *	37.6 ± 3.6 *	28.4 ± 2.1	29.3 ± 6.5
Body weight [kg]	80.4 ± 4.1	79.5 ± 13.8	84.1 ± 9.8 *	82.1 ± 7.8	81.5 ± 2.0
Height [cm]	177.9 ± 3.5	179.3 ± 6.7	179.7 ± 6.3	177.7 ± 6.3	178.6 ± 0.9
BMI	25.4 ± 1.6	24.8 ± 1.4	26.3 ± 1.2 *	26.2 ± 1.4 *	25.7 ± 1.4

* Statistically significant difference at *p* < 0.05.

**Table 4:** Values of the average daily energy expenditure of a missile frigate’s crew, who indicated an average body weight of 80.4 kg, during a cruise.

Time	Activity	Duration of the Activity [min]	Energy Expenditure [kcal/min/kg b. m.]	Energy Expenditure of the Activity [kcal]
3^15^–3^50^	Night patrol	35	0.0433	121.8 ± 7.2
3^50^–4^00^	Night watch briefing	10	0.0310	24.9 ± 2.2
4^00^–6^00^	Change of sea watch	120	0.0310	299.1 ± 14.1
6^00^–6^10^	Wake up and preparation for morning exercises	10	0.0475	38.2 ± 2.6
6^10^–6^30^	Morning exercises	20	0.0706	113.5 ± 3.3
6^30^–6^50^	Morning wash up	20	0.0595	95.7 ± 2.6
6^50^–7^20^	Cleaning the ship	30	0.0425	102.5± 3.1
7^20^–7^50^	Breakfast	30	0.0412	99.4 ± 1.1
7^50^–8^00^	New watch briefing	10	0.0310	24.9 ± 3.3
8^00^–11^30^	Training and ship service	210	0.0381	643.3 ± 22.2
11^30^–12^00^	Preparation for lunch	30	0.0475	114.6 ± 4.6
12^00^–12^30^	Lunch	30	0.0412	99.4 ± 8.1
12^30^–14^00^	Break after lunch	90	0.0202	146.2 ± 4.1
14^00^–16^00^	Training and ship service	120	0.0381	367.6 ± 21.2
16^00^–16^20^	1st dinner	20	0.0412	66.2 ± 13.6
16^20^–16^30^	Briefing and change of watch	10	0.0310	24.9 ± 2.2
16^30^–17^50^	Time at the ship commander’s disposal	80	0.0214	137.6 ± 11.6
17^50^–18^15^	Briefing and change of watch	25	0.0310	62.3 ± 3.6
18^15^–19^30^	Auxiliary work in the ship’s kitchen	75	0.0614	370.2 ± 17.5
19^30^–20^00^	Free time	30	0.0360	86.8 ± 5.3
20^00^–20^20^	2nd dinner	20	0.0412	66.2 ± 6.6
20^20^–20^30^	Watch briefing	10	0.0310	24.9 ± 3.3
20^30^–20^45^	Change of sea watch	15	0.0276	33.3 ± 2.2
20^45^–21^30^	Evening patrol	45	0.0433	156.6 ± 42.1
21^30^–21^50^	Evening toilet	20	0.0595	95.7 ± 12.3
21^50^–22^00^	Night watch briefing	10	0.0310	24.9 ± 3.2
22^00^–22^15^	Change of sea watch	15	0.0276	33.3 ± 2.2
22^15^–3^15^	Sleep	300	0.0166	400.4 ± 22.6
**Daily energy expenditure**		**1440**		**3874 ± 248**

**Table 5:** Values of the average daily energy expenditure of a school sailing ship’s crew, who indicated an average body weight of 79.5 kg, during a cruise.

Time	Activity	Duration of the Activity [min]	Energy Expenditure [kcal/min/kg b. m.]	Energy Expenditure of the Activity [kcal]
3^15^–3^50^	Night patrol	35	0.0433	120.5 ± 22.6
3^50^–4^00^	Night watch briefing	10	0.0310	24.6± 6.6
4^00^–6^00^	Change of sea watch	120	0.0310	295.7 ± 22.1
6^00^–6^10^	Wake up and preparation for morning exercises	10	0.0475	37.8 ± 11.3
6^10^–6^30^	Morning exercises	20	0.0706	112.2 ± 13.7
6^30^–6^50^	Morning wash up	20	0.0595	94.6 ± 6.4
6^50^–7^20^	Cleaning the ship	30	0.0425	101.4 ± 13.2
7^20^–7^50^	Breakfast	30	0.0412	98.3 ± 8.9
7^50^–8^00^	New watch briefing	10	0.0310	24.6 ± 6.6
8^00^–11^30^	Training and ship service	210	0.0456	761.3 ± 33.9
11^30^–12^00^	Preparation for lunch	30	0.0475	114.0 ± 10.6
12^00^–12^30^	Lunch	30	0.0412	98.3 ± 8.1
12^30^–14^00^	Break after lunch	90	0.0202	144.5± 23.5
14^00^–16^00^	Training and ship service	120	0.0456	435.0± 38.2
16^00^–16^20^	1st dinner	20	0.0412	65.5 ± 13.3
16^20^–16^30^	Briefing and change of watch	10	0.0310	24.6 ± 6.3
16^30^–17^50^	Time at the ship commander’s disposal	80	0.0214	136.1 ± 21.7
17^50^–18^15^	Briefing and change of watch	25	0.0310	61.6 ± 11.6
18^15^–19^30^	Auxiliary work in the ship’s kitchen	75	0.0614	368.4± 56,7
19^30^–20^00^	Free time	30	0.0360	85.8± 13.7
20^00^–20^20^	2nd dinner	20	0.0412	65.5 ± 17.1
20^20^–20^30^	Watch briefing	10	0.0310	24.6 ± 6.3
20^30^–20^45^	Change of sea watch	15	0.0276	32.9 ± 5.2
20^45^–21^30^	Evening patrol	45	0.0433	154.9 ± 33.3
21^30^–21^50^	Evening toilet	20	0.0595	94.6 ± 17.2
21^50^–22^00^	Night watch briefing	10	0.0310	24.6 ± 6.3
22^00^–22^15^	Change of sea watch	15	0.0276	37.0 ± 6.6
22^15^–3^15^	Sleep	300	0.0166	237.5 ± 18.8
**Daily energy expenditure**		**1440**		**4031 ± 436**

**Table 6:** Values of the average daily energy expenditure of a missile frigate’s crew, who indicted an average body weight of 80.4 kg, during their stay in a port.

Time	Activity	Duration of the Activity [min]	Energy Expenditure [kcal/min/kg b. m.]	Energy Expenditure of the Activity [kcal]
6^00^–6^10^	Wake up and preparation for morning exercises	10	0.0475	38.2 ± 2.6
6^10^–6^30^	Morning exercises	20	0.0706	113.5 ± 17.6
6^30^–7^00^	Morning wash up	30	0.0595	143.5 ± 22.8
7^00^–7^20^	Breakfast	20	0.0412	66.2 ± 12.1
7^20^–7^40^	Cleaning the ship	20	0.0624	100.3 ± 18.3
7^40^–7^50^	New watch briefing	10	0.0310	24.9 ± 4.7
7^50^–8^00^	Raising the flag	10	0.0310	24.9 ± 5.3
8^00^–8^35^	Overview and rotated mechanisms	35	0.0433	121.8± 38.8
8^35^–12^15^	Activities on the ship	220	0.0433	765.9 ± 44.5
12^15^–13^15^	Lunch break	60	0.0412	198.7 ± 23.6
13^15^–15^00^	Scheduled classes	105	0.0433	365.5 ± 36.8
15^00^–15^20^	Roll call	20	0.0310	49.8 ± 8.2
15^20^–16^00^	Cleaning the ship	40	0.0425	136.7 ± 27.9
16^00^–18^00^	Free time	120	0.0214	206.5 ± 33.1
18^00^–18^30^	Dinner	30	0.0412	99.4 ± 11.2
18^30^–21^00^	Free time	150	0.0214	258.1 ± 28.9
21^00^–21^30^	Cleaning the ship	30	0.0624	150.5 ± 9.6
21^30^–22^00^	Evening toilet	30	0.0595	143.5 ± 11.8
22^00^–6^00^	Sleep	480	0.0166	640.6 ± 26.9
**Daily energy expenditure**		**1440**		**3648 ± 332**

**Table 7:** Values of the average daily energy expenditure of a school sailing ship’s crew, who indicted an average body weight of 79.5 kg, during their stay in port.

Time	The Name of the Activity	Duration of the Activity [min]	Energy Expenditure [kcal/min/kg b. m.]	Energy Expenditure of the Activity [kcal]
6^00^–6^10^	Wake up and preparation for morning exercises	10	0.0475	37.8 ± 6.2
6^10^–6^30^	Morning exercises	20	0.0706	112.2 ± 29.3
6^30^–7^00^	Morning wash up	30	0.0595	141.9 ± 21.5
7^00^–7^20^	Breakfast	20	0.0412	65.5 ± 11.6
7^20^–7^40^	Cleaning the ship	20	0.0624	99.2 ± 18.8
7^40^–7^50^	New watch briefing	10	0.0310	24.6 ± 5.1
7^50^–8^00^	Raising the flag	10	0.0310	24.6 ± 5.0
8^00^–8^35^	Overview and rotated mechanisms	35	0.1199	333.6 ± 42.1
8^35^–12^15^	Activities on the ship	220	0.0456	797.5± 53.7
12^15^–13^15^	Lunch break	60	0.0412	196.5± 21.2
13^15^–15^00^	Scheduled classes	105	0.0456	380.6 ± 33.1
15^00^–15^20^	Roll call	20	0.0310	49.3 ± 9.7
15^20^–16^00^	Cleaning the ship	40	0.0425	135.2 ± 21.6
16^00^–18^00^	Free time	120	0.0214	204.2 ± 33.1
18^00^–18^30^	Dinner	30	0.0341	98.3 ± 15.5
18^30^–21^00^	Free time	150	0.0214	255.2 ± 34.3
21^00^–21^30^	Cleaning the ship	30	0.0624	148.8 ± 38.7
21^30^–22^00^	Evening toilet	30	0.0595	141.9 ± 26.8
22^00^–6^00^	Sleep	480	0.0166	633.5 ± 33.7
**Daily energy expenditure**		**1440**		**3380 ± 461**

**Table 8:** Energy load of an officer with an average body weight of 84.1 ± 9.8 kg during a 12 h service in a Border Guard Unit.

n = 89	Maritime Border Guard Unit							
		x	±	SD	Median	Min	÷	Max
Energy expenditure	time [h]	15.6	±	6.1	11.7	7.2	÷	23.3
	kcal/h	142	±	50	138	61	÷	230
	kcal/min	2.36	±	0.83	2.30	1.02	÷	3.84
	kcal/h/kg b. m.	2.13	±	0.94	1.92	0.67	÷	3.89
	kcal/min/kg b. m. b.m. mc	0.036	±	0.016	0.032	0.011	÷	0.065
	kcal measured	2100	±	959	1661	954	÷	4193
Pulse	max	149	±	32	140	107	÷	220
	min	53	±	7	53	42	÷	68
	average	82	±	11	78	69	÷	102
**Total**	**Kcal/12 h**	**1703**	**±**	**599**	**1657**	**735**	**÷**	**2762**

**Table 9:** Energy load of an officer with an average body weight of 82.1 kg during a 12 h service at the Kashubian Border Guard Division.

n = 21	Kashubian Border Guard Division							
		x	±	SD	Median	Min	÷	Max
Energy expenditure	time [h]	25.3	±	0.2	25.3	25.2	÷	25.6
	kcal/h	98	±	39	73	69	÷	153
	kcal/min	1.64	±	0.64	1.22	1.15	÷	2.54
	kcal/h/kg mc	1.35	±	0.66	0.94	0.84	÷	2.28
	kcal/min/kg mc	0.023	±	0.011	0.016	0.014	÷	0.038
	kcal measured	2482	±	960	1868	1739		3838
Pulse	max	146	±	18	151	122	÷	166
	min	55	±	9	61	43	÷	62
	average	77	±	6	79	68	÷	83
**Total**	**Kcal/12 h**	**1178**	**±**	**462**	**876**	**826**	**÷**	**1830**

**Table 10:** Energy and nutritional values of food rations used in the feeding of ship crews.

	Missile Frigate		School Sailing Ship	
	Planned Food Ration	Food Ration Given for Consumption	Planned Food Ration	Food Ration Given for Consumption
Energy value (kcal)	4430.9 ± 322.6	4120.0 ± 300.8 *^,●^	4234.7 ± 419.6	3520.9 ± 365.0 *
Protein content (g)	158,3 ± 17.4	149,3 ± 17.9	163.0 ± 14.7	108.2 ± 13.9 *
Fat content (g)	201.0 ± 34.6 ^●^	147.4 ± 23.5 *	167.5 ± 28.6	108.7 ± 21.3 *
Carbohydrates content (g)	497.2 ± 53.3 ^●^	547.5 ± 53.3 *^,●^	518.7 ± 64.2 ^●^	527.5 ± 54.2

* Statistically significant difference from the planned ration (*p* < 0.05). ^●^ Statistically significant difference from the food standard (*p* < 0.05).

## Data Availability

The data presented in this study are available upon request from the corresponding author. The data are not publicly available due to privacy and ethical restrictions.
